# Autointoxication and historical precursors of the microbiome–gut–brain axis

**DOI:** 10.1080/16512235.2018.1548249

**Published:** 2018-11-27

**Authors:** Manon Mathias

**Affiliations:** School of Modern Languages and Cultures, University of Glasgow, Glasgow, UK

**Keywords:** Microbiome–gut–brain axis, mental health, autointoxication, history, depression, Bouchard

## Abstract

This article focuses on autointoxication, a discredited medical theory from the late nineteenth century that provides important points of reflection for today’s research on the role of microbes in the human gut for mental health. It considers how the theory of autointoxication, which came into great prominence amongst physicians and the general public worldwide, fell from grace by the middle of the twentieth century, and briefly asks why studies of the human microbiome are now back in vogue. It departs from earlier articles on the topic firstly by arguing that autointoxication theory was especially prevalent in France, and secondly by focusing on the application of this theory to mental health. Bringing to light medical treatises and theses from this period which have so far remained unexamined, it shows that examining the development and reception of medical theories form the past can help us today in understanding both the pitfalls and promise of research in this area.

## Introduction

As recently as 2005, the suggestion that bacteria in the human gut could be a factor in mental health was highly speculative, even contentious [1]. Even in 2011, it was still located in the hypothetical realm [2]. Almost a decade later, more clinical trials are needed on humans, as is pointed out in recent reviews [3,4], and the precise nature of the process is still not known. But numerous studies have demonstrated clear links between intestinal microbiota and mood, behaviour, and cognitive impairment [5–8] and such findings could have radical implications for the treatment of neuropsychiatric disorders. However, the idea that bacteria in the human gut could play role in mental health is not revolutionary, and this article argues that the prehistory of this area provides important context for present-day research. Specifically, it will be shown that the heightened interest in ‘autointoxication theory’ in nineteenth-century France provides important points of reflection for contemporary work on the gut–brain–microbiome connection. The article provides an important corrective to common views of autointoxication as a mere quack theory grounded in nineteenth-century obsessions with constipation. It shows instead that many of the physicians who wrote about autointoxication were in fact interested in microbe–mind interactions, whilst those associated with debunking autointoxication were the most resistant to recognising the potential role of intestinal bacteria in mental health.

## Autointoxication theory takes off

Following on from Louis Pasteur’s discoveries in the sphere now termed bacteriology, researchers in the 1880s and 1890s became highly interested in the significance of microbes located in the digestive system. Specifically, European physicians pondered about the influence of these microbes on human health through the internal processes of ‘putrefaction’ [9]. There were several claims to precedence in this area. For example, some have referred to Dr Robert Bell as an early developer of what came to be known as autointoxication theory, since he claimed that putrid ‘fluid’ absorbed from the large intestine led to a form of blood-poisoning [10]. But as Robert Hudson notes, Bell did not indict bacterial toxins specifically [11].

Much of the early groundwork for the theory was laid out by German physicians. The Prussian specialist in internal medicine, Hermann Senator, speculated in a brief article of 1868 that ‘self-infection’ through bacteria in the intestines could lead to disease [12]. The work carried out by German physician and medical writer Ludwig Brieger on ‘ptomaines’ also proved influential: in his three-volume *Über Ptomaine* (1885–1886), Brieger analysed the chemical processes that occurred during the ‘putrefaction’ of proteins within the human intestine. He referred to the basic products formed during this process as ‘ptomaines’ (from the ancient Greek *πτῶμα* for fallen body, or corpse) [13] and argued that their absorption was harmful to the human body [14,15].

But it was French physician Charles Bouchard’s theory of ‘autointoxication’ which did the most to stimulate research on the role of intestinal microbes in health. Bouchard noted in his famous *Lectures on Autointoxication in Disease* (1887) that ‘man is inhabited, most considerably in his digestive tract, by lower organisms’, and if these organisms were not properly eliminated, or if too many of them were produced, they could cause what he termed internal ‘poisoning’, leading to disease. Thus ‘man […] carries within himself the cause of many illnesses’, he stated [16]. Bouchard’s theory was notoriously vague, but it was to have remarkably wide currency, particularly in France and Germany, and also in the US, largely spearheaded by French and German-trained physicians [17].

**Figure 1.**
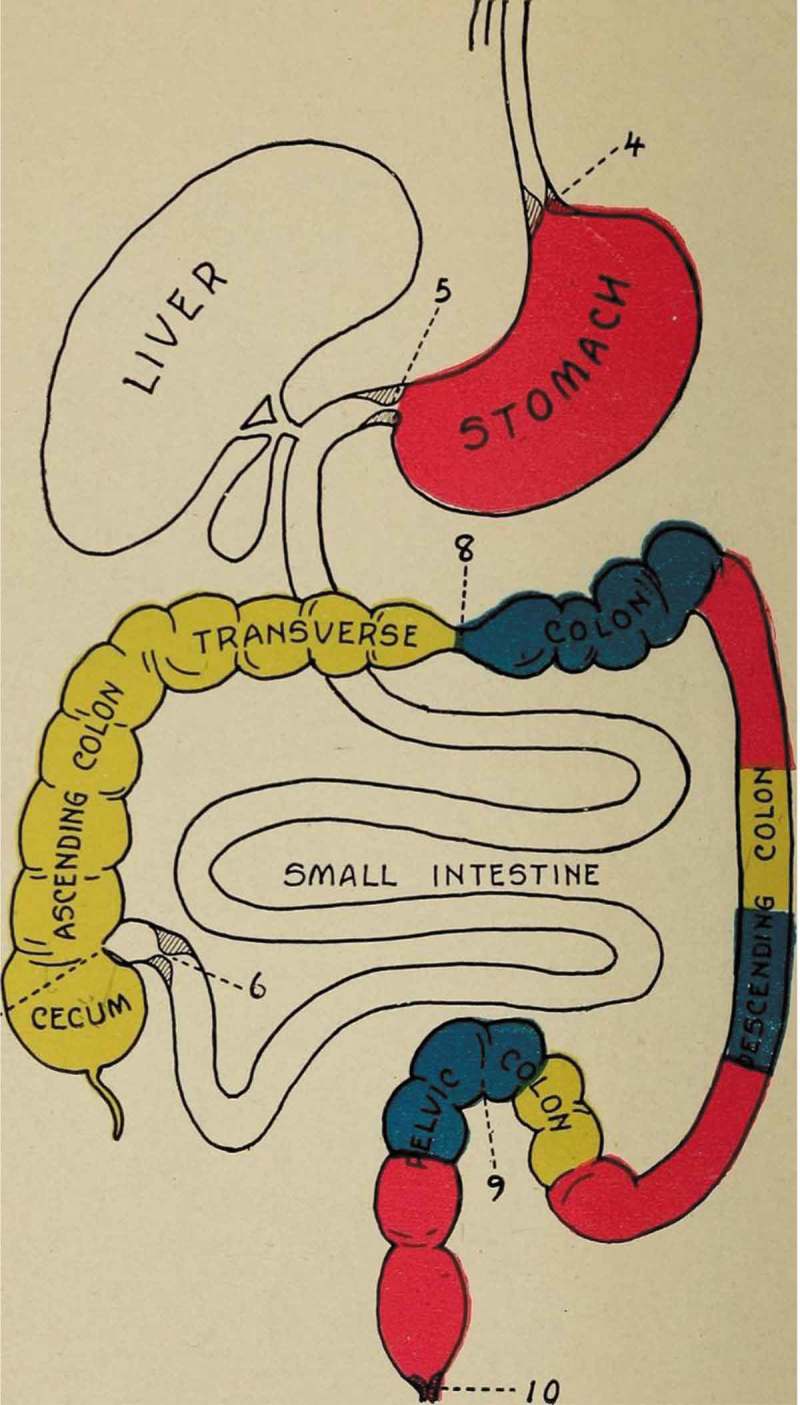
Kellogg, John Harvey, *The Itinerary of a Breakfast; a Popular Account of the Travels of a Breakfast Through the Food Tube And of the Ten Gates and Several Stations through which it Passes, Also of the Obstacles which It Sometimes Meets* (1920) by Kellogg, John Harvey, 1852–1943 [No restrictions], via Wikimedia Commons https://archive.org/stream/itineraryofbre00kell/itineraryofbre00kell#page/n139/mode/1up.

The American health reformer, John Harvey Kellogg, for example, popularized the theory in his bestselling *Autointoxication or Intestinal Toxemia* (1919) where he referred to the concept as that of ‘the French school’. Some still swear by these theories, as seen in the republication of Kellogg’s text in 2006 [18]. Kellogg claimed in 1919 that ‘at the present time there are few up-to-date medical men, who do not recognize the close relation between intestinal stasis […] and a long list of chronic disorders’ [19]. His volume enthusiastically describes methods for dealing with ‘cases requiring change of the intestinal flora’, methods that he claimed to have ‘successfully employed’ to treat thousands of patients at his Battle Creek Sanitarium. Such is his interest in the topic, he devotes further attention to autointoxication in his next publication, *The Itinerary of a Breakfast* (1920), where he asserts that ‘intestinal toxaemia or autointoxication is the most universal of all maladies, and the source of autointoxication is the colon with its seething mass of putrefying food residues’, outlined in a coloured illustration (Figure 1).

## Autointoxication: a swift fall from grace

**Figure 2.**
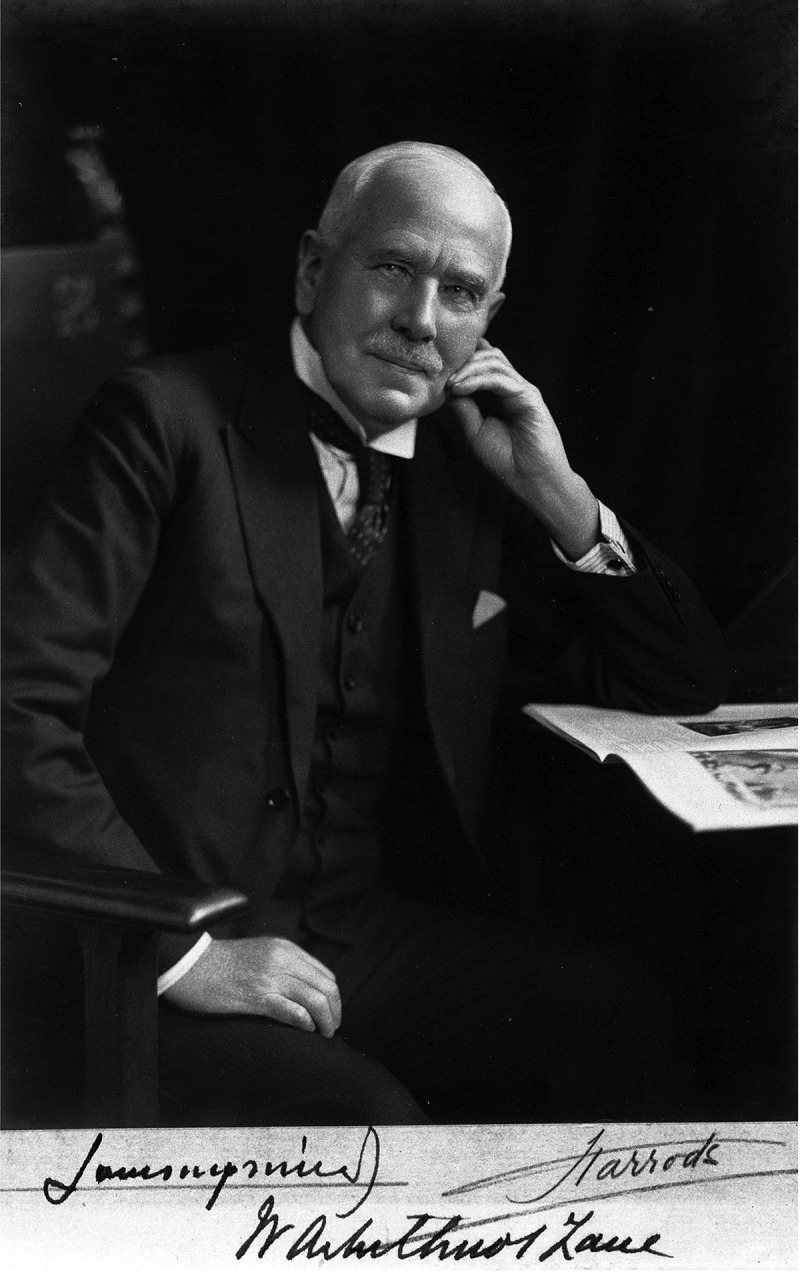
Autographed portrait of Sir W. Arbuthnot Lane, from an original photograph by Harrods. Lent by the director, Wellcome Medical Museum. Wellcome Collection.

Despite the intense medical and popular interest in autointoxication at the turn of the century, however, serious research on the topic was short-lived, and it largely fell out of favour amongst scientists and most practitioners by the 1930s. There are numerous reasons behind this dramatic decline, and many have stressed the problems and failings of the model [11,17,20–22]. One of the reasons that autointoxication became discredited, for example, was that certain established physicians took up the theory in the early twentieth century as a basis for extreme and unnecessary treatments. The most famous example is that of Scottish physician, Sir William Arbuthnot Lane (Figure 2), who regularly performed colectomies as a way of treating cases of intestinal stasis [23]. Lane’s purported success motivated further surgeons, such as Henry Cotton and John Draper in the US, to perform risky, invasive procedures based on little evidence and leading to little respite and at times, death [24]. Such radical approaches further undermined serious interest in intestinal bacteria.

However, it is rarely pointed out that Lane was mostly interested in problematic abdominal positioning rather than the role of microbes in the gut. Lane believed that the upright biped position of human beings was harmful for the digestive system, as it placed stress on the colon and thus harmed both its form and function [25,26]. It was not Lane’s adherence to autointoxication theory that led to opposition, but rather his doubtful ideas about the positioning of the bowels and his repeated removal of patients’ colons as an extreme response to what he saw as the resulting problems.

This presents one of the other reasons behind autointoxication’s fall from grace: the fact that it was regularly conflated with other conditions and theories, including infarctus (impacted faeces, a theory associated with Johann Kampf, 20), constipation, distention, and visceroptosis. Charles Bouchard’s early interest in abdominal distention, for example, was one of the reasons behind his opponents’ initial dismissal of his broader theory of autointoxication [27]. Autointoxication theory was also (and still is) often confused with visceroptosis, a condition associated with physician Frantz Glénard which broadly referred to the displacement or prolapse of the bowels [28]. Baron and Sonnenfeld’s 2002 article, for example, examines visceroptosis and autointoxication concomitantly and dismisses both as ‘nonexistent disease entities’ [17].

**Figure 3.**
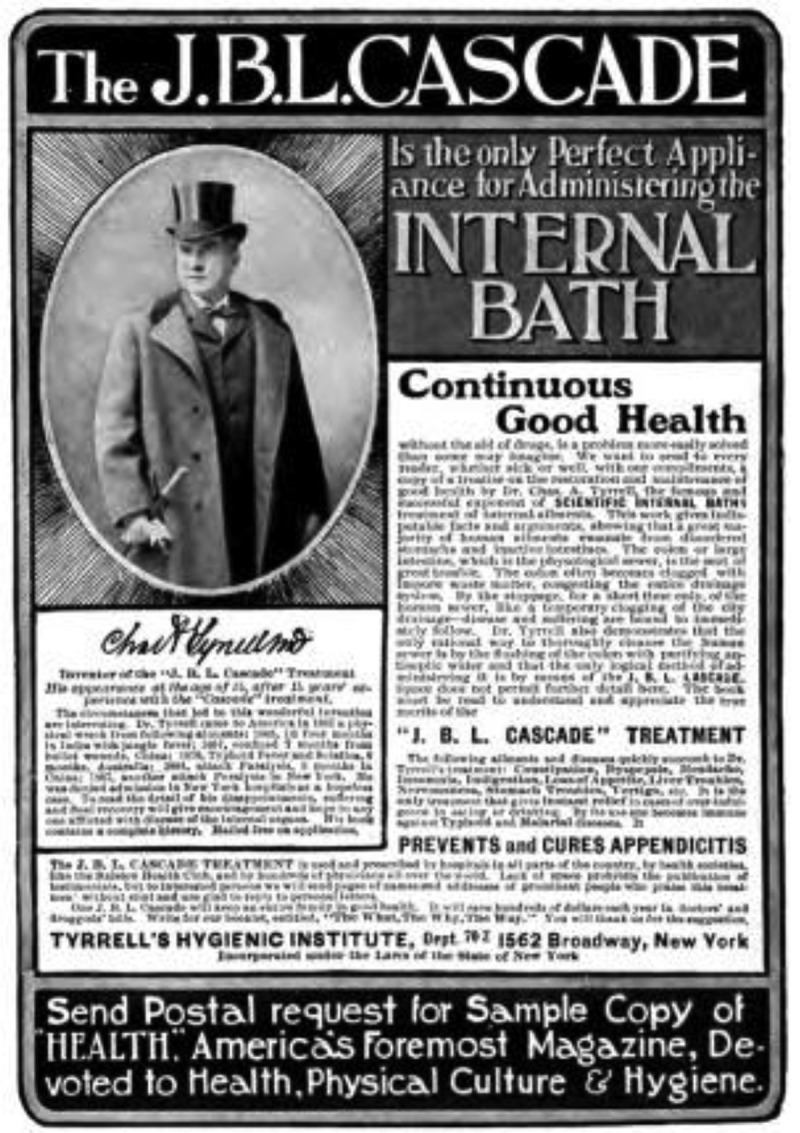
An advertisement for the J.B.L. Cascade, *Health: a Home Magazine dedicated to Physical Culutre and Hygiene*, December 1905 issue. https://archive.org/stream/healthahomemaga00unkngoog#page/n492/mode/2up Copyright expired, originally printed prior to 1923 in the US.

A further reason behind autointoxication theory’s decline was that it quickly became associated with quackery. Alongside the legitimate scientific interest in the effects of intestinal bacteria on health, alternative practitioners and charlatans were alert to the financial possibilities offered by the idea that cleaning out the colon could instantly improve wellbeing [22]. Opportunistic entrepreneurs appropriated the theory in order to sell dubious therapies based on unfounded claims. Charles A. Tyrrell’s syringe enema, ‘the Cascade’, for example, purported to cure a host of maladies, all of which Tyrrell attributed to so-called intestinal poisoning [22] (Figure 3).

## Autointoxication and its links with today’s research

It is not surprising that unscrupulous individuals regularly used hyperbolic language to discuss cures for autointoxication as a means of selling their ultimately useless products. In his *The Royal Road to Health* (1894), for instance, Tyrrell claimed that the cause of all disease was ‘the retention of waste matters in the system’, and thus ‘the average colon is a fertile breeding ground for all kinds of poisonous germs’ [29]. Luckily, he could offer a method of treatment which he ‘unhesitatingly’ affirmed would be found ‘so simple, so inexpensive and so obviously based on common sense and true hygienic principles, that the thoughtful reader cannot fail to give it his unqualified endorsement, and will be lost in wonder that any one should fail to adopt it, when made acquainted with its simplicity and its marvellous results.’ This wondrous instrument was the ‘J.B. L. Cascade’, a mechanical appliance named after the words ‘Joy, Beauty, Life, which aptly indicate its purpose and effects, for we confidently claim that its use will infallibly confer these three great blessings, it being the one safe and sanative method of regaining and preserving health.’ Tyrrell’s overly self-assured phrasing (‘unhesitatingly’, ‘unqualified’, ‘confidently’, ‘infallibly’) and quasi-religious language (‘wonder’, ‘marvellous’, ‘blessing’) are signals of his misleading purposes.

But exaggerated claims were not confined to charlatans. On a broader level, the vague nature of autointoxication theory made it amenable to all forms of conditions and it was particularly useful as a diagnosis for the myriad unexplained and poorly understood symptoms of ‘hypochondria’, ‘neurasthenia’ or ‘melancholia’, which would today be catalogued as co-morbid gastrointestinal and anxiety-related disorders. Autointoxication thus met a need to explain such symptoms with no identifiable source, and it became a catch-all diagnosis.

This sense that autointoxication could be seen as the cause of all disease was not considered problematic but instead made it highly attractive: its lure can for instance be detected in French psychiatrist Emmanuel Régis’s allusion to experimentation in this area as ‘*seductive* and full of promise of immediate scientific success’ (30. added emphasis). Referring to the psychic symptoms which often accompanied gastrointestinal illness, Régis claimed that Charles Bouchard had ‘shone new light on these symptoms’ [31].

Such comments remind us of today’s excitement surrounding the human microbiota: although most researchers scrupulously qualify their findings and acknowledge the preliminary nature of work in this area, the notion of the gut as the key to health is proving to be highly attractive. For Emeran Mayer, ensuring that our gut–microbiota–brain interactions are ‘functioning at peak effectiveness’ can help us achieve ‘optimal health’ [32]. A recent article in *Frontiers in Genetics* argues that ‘the human microbiome has emerged as the crucial moderator in the interactions between food and our body’, and thus research in this area has moved from a marginalised position to become ‘a beacon of hope with great potential and many possibilities’ [33]. Once again the image of light cast over shadows suggests that we have finally reached an explanation for hitherto mysterious maladies.

A potential explanation behind the enthusiasm surrounding the microbiome–gut–brain axis today, in contrast with the scorn poured on such connections in the early twentieth century, is the shift in attitude towards the environment and the rise of ‘green studies’, as argued by Funke Iyabo Sangodeyi [34]. This point is also raised by Nitin and Amisha Ahuja, who comment on the ecological metaphors regularly used to describe the human microbiome [35]. Ed Yong’s *I Contain Multitudes* (2017), for example, refers to the ‘ecosystems’ inside us [36], and many researchers now refer to the human body in such ecological terms [37,38]. Since we are now much more aware of the inextricable bonds between human, animal and mineral life, the notion that we also have a symbiotic relationship with non-human elements within our own bodies is becoming increasingly logical.

On one hand, current use of such ecological frameworks and an emphasis on ‘traditional’ or ‘natural’ lifestyles to discuss the functioning human microbiome (seen as out of sync with twenty-first-century living) is an apt and logical progression from the early theory of autointoxication, which was also associated with the dangers of modern, industrialised society such as pollution, chaotic eating habits, and psychological and digestive disorder [35,39]. But on the other hand, there are major differences between these two research areas today, not least on the level of development, since gut–brain–microbiome research is still at an early stage, whereas environmental science is highly advanced, and the evidence for climate change and the effects of human lifestyles on the planet is vast. Moreover, we need to consider the specificity of gut–brain research in the context of mental health, which is where autointoxication theory proves most noteworthy of our interest.

## Autointoxication theory and mental health

It has often been claimed that the theory of autointoxication was definitively ‘disproven’ [22] by American physicians Walter Alvarez and Arthur Donaldson in the 1920s. Chen and Chen, for example, affirm that these physicians’ ‘decisive experiments’ [20] invalidated intestinal autointoxication, and others also make similar affirmations [40]. Edzard Ernst, for example, referring to Donaldson’s work, also claims that ‘rigorous scientific investigation into the theory of autointoxication’ meant that ‘the hypothesis was soon found to be wrong’ [21]. Alvarez and Donaldson’s research showed that typical symptoms of autointoxication – loss of appetite, mental sluggishness, headaches and depression – were caused by constipation rather than chemical forces such as the absorption of poisons. They therefore challenged the importance of intestinal microbes in these symptoms.

As Bested et al. have recently shown, however, the notion that Alvarez and Donaldson’s research was both rigorous and decisive is a ‘modern myth’ [24]. Most importantly for this article, Alvarez and Donaldson focused on constipation rather than autointoxication itself: their experiments were carried out on patients suffering from chronic constipation; healthy individuals who were made to refrain from defecating for several hours; or individuals whose rectums were packed with cotton [22,24]. What they showed was that the symptoms were caused by ‘mechanical distention and irritation of the lower bowel by fecal masses’ rather than ‘poisoning’ through bacteria [22]. The hypothesis that bacteria in the human intestines could have an impact on mental health, on the other hand, was never disproven, since it was not subjected to rigorous trials. Therefore, these two physicians, who are routinely considered to have invalidated autointoxication, were unwilling to acknowledge or even to consider the potential role of intestinal bacteria in mental health.

The assumption that autointoxication theory can be dismissed wholesale, however, due to its presumed association with other complaints, continues: Micaela Sullivan-Fowler, for example, conflates autointoxication with constipation and colonic irregularity in general [22], and Ernst presents it as ‘a triumph of ignorance over science’ by equating it with extreme treatments such as colonic irrigation [21]. J. L. Smith also asserts that ‘chronic intestinal stasis was a pseudodisease, an aberration of medical progress’ [40]. James C. Whorton’s excellent work on the social and cultural importance of ‘inner hygiene’ repeatedly conflates autointoxication with constipation [41]: he affirms, for example, that ‘the constipated person, French physician Charles Bouchard declared, “is always working toward his own destruction; he makes continual attempts at suicide by intoxication.”’ [39]. Although Bouchard did indeed make this comment about man working towards his own destruction (through the organisms in his gut), the comment was not made in relation to constipation. Rather, Bouchard affirmed that constipation could be a form of protection against autointoxication, since constipation assumes that everything that can be absorbed has been absorbed, and whereas there is a risk of intoxication in the first stage of constipation, by the second stage, intoxication is no longer in operation [16].

Such examples give an indication of the early reasons behind the discrediting of microbes’ importance for mental health research, and suggest pitfalls for researchers to avoid: conflation with broader conditions and symptoms; recruitment by individuals and organisations driven by commercial interests; misappropriation of the theory to sell products based on unfounded claims; and exaggerated, overhyped promises. Such factors enabled commentators in the first half of the twentieth century to dismiss the potential links between gut bacteria and the mind before sufficient human evidence could even be established.

But if we look at the early research that specifically explored the role of intestinal bacteria in psychiatric conditions, autointoxication emerges as a fruitful area of inquiry. Bouchard’s lectures tied in with the findings of many physicians across the Western world regarding the role of bacteria in causing disease. But his theory of autointoxication took particular hold in France, specifically in relation to mental health. It is beyond the scope of this article to consider the compelling cultural factors behind France’s strong involvement with this theory (unlike in Britain where take-up was lower) [17]. But we can note that, within the realm of medical history, France had a particularly strong tradition of linking the state of the digestive system with psychiatric health or what was termed ‘la santé morale’.

Philosopher and physiologist Pierre-Jean-Georges Cabanis (1757–1808), for example, argued that moral life (or the life of the mind) was not only affected by impressions received by the senses but also by those received from the viscera. He stressed the stomach’s influence on the nervous system, and especially its immediate impact on the brain [42]. The military physician, François-Joseph-Victor Broussais, famously argued that all passions and mental states are caused by visceral sensation, and in his epigastric theory, hypochondria and similar conditions such as neurasthenia and melancholia were all located in the viscera [43]. Philippe Pinel, often seen as the ‘father of psychiatry’ in France [44], also argued that the seat of mental illness was to be found in what he termed the ‘epigastric region’, and he saw symptoms such as constipation or the tightening of the stomach as early symptoms of mania [45]. Mental illness was therefore partly visceral in origin [44]. Pinel’s student, the highly influential psychiatrist, Esquirol, also emphasised visceral lesions in cases of insanity in his thesis of 1805 [46].

## Early pioneers: autointoxication and psychiatric disorders

Physicians from the early nineteenth century often focused on the nerves as the key connection between the gut and the brain, and portrayed the connection as a form of ‘sympathy’ [47] between the two regions or a process of ‘irradiation’ from stomach to brain [48]. This is in contrast with many scientists and practitioners from the end of the century who focused on bacteria in the gut as the essential factor. Emmanuel Régis was a pioneer in this regard. Régis became particularly interested in the potential of this connection in the 1880s through his observations of psychiatric patients. He noticed that their condition improved after they had received treatment for gastric symptoms, which suggested to him that the bacteria in their intestines might be intervening in their mental balance. He himself did not publish on this until 1893, but before that point he encouraged other researchers to investigate.

Bested et al. have highlighted autointoxication as an antecedent for current gut–brain–microbiome research, but the first original paper on autointoxication and melancholia that they refer to is physician Daniel R. Brower’s article in the *Journal of the American Medical Association* in 1898 [24,49]. The first publication suggesting a link between autointoxication and mental health in fact seems to have been *de l’influence Des phénomènes d’auto-intoxication et de La dilatation de l’estomac dans les formes dépressives et mélancoliques* of 1891, an extract of a presentation given by physician Antónoio Mario de Bettencourt Rodrigues at a mental health congress in Paris, August 1889. Rodrigues was a Portuguese physician, but he was based at the Faculty of Medicine in Paris and he trained under French psychologist Georges Dumas.

Rodrigues argued that one of the main factors that could trigger mental disorders was gastrointestinal autointoxication, particularly in the case of depression and melancholia. He cited Emmanuel Régis and Charles Bouchard as his key inspirations in this regard [50]. Given his many experiences of mental patients improving after a change in diet combined with the elimination of toxins in the gut, Rodrigues argued that there was all the reason to believe that autointoxication was the cause of the psychiatric disorders in these patients. Rodrigues admitted to having little proof to support his claims – the task of compiling such evidence would fall to François-André Chevalier-Lavaure, ‘the first experimental scientist to apply Bouchard’s ideas to the realm of mental pathology’, according to a later medical practitioner [51].

**Figure 4.**
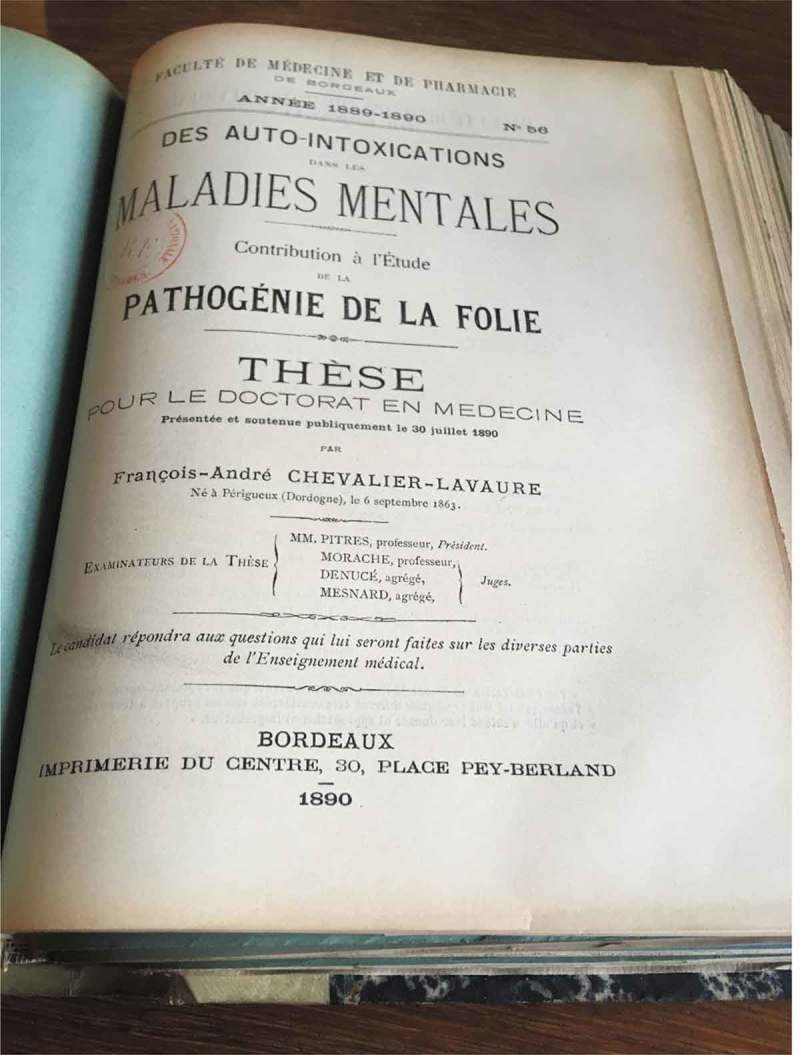
Medical thesis by Chevalier-Lavaure, Bibliotheque Nationale de France, picture taken Manon Mathias, 2 July 2018. Copyright expired.

Chevalier-Lavaure, in his doctoral thesis of 1890 (Figure 4), explained that there are always bacteria in the human organism, but digestive problems sometimes lead to an augmentation in the level of bacteria and a qualitative and quantitative change in their toxicity [52]. Insufficient elimination of these toxic products means that they can make their way into the patient’s blood and, in this way, affect the brain. Although we now know that this argumentation in itself is false, Chevalier-Lavaure did note an improvement in psychiatric patients after the treatment of digestive conditions. He posited that this was due to the fact that harmful bacteria had been prevented from forming, but experiments were needed to prove this.

Chevalier-Lavaure provides a list of case studies involving patients suffering from psychiatric conditions. His experiments showed that digestive problems preceded their psychiatric disturbances, and when these problems were treated, the mental disturbance disappeared. He acknowledged the important role played by heredity in the development of such conditions, but he affirmed that a trigger was needed to set off this predisposition, ‘and this trigger is autointoxication.’ Mental disturbance, he stated, is caused by ‘the effects produced on the brain by the poisons released by an unhealthy organ in which the nutritive process is disturbed.’ Chevalier-Lavaure admitted that he had little means of confirming the so-called ‘toxicity’ of the contents of the bowel, but he could measure what came out: he thus used patient’s urine as a means of ascertaining the level of ‘toxins’ in the patients’ system [52].

Chevalier-Lavaure did not claim to have offered definitive proof, and some of his suggestions have proved to be erroneous. But his central aim, inspired by the suggestions made by Régis and Bettencourt Rodigues, of drawing attention to the relations between mental disorders and ‘toxins’ in the digestive system (not digestive problems in general, but ‘the most important factor: autointoxication’), was highly prescient. His work thus shows that not all of autointoxication was mere quackery, and instead some of the earliest researchers took a serious and careful interest in microbe–mind interactions.

As recently as 2003, Ann Dally considered autointoxication as no more than a ‘fashionable’ but discredited theory. Focusing on the resulting treatments, she presents it as a theory which supports the ‘modern’ model of the ‘body as machine with working parts, quite separate from the mind’ [53]. But as this paper has shown, researchers in France at the end of the nineteenth century were, on the contrary, applying ideas about the role of gut bacteria in health and disease to rethink the disorders of the mind. Such was the interest in this area of research in France that the 1893 French Congress of Psychological Medicine held in La Rochelle featured a panel session dedicated to the topic of intestinal autointoxication in mental health.

## Treatment

In terms of treatment, Chevalier-Lavaure suggested that in future, patients’ faecal matter should be examined in order to find out its toxicity, and the harmful bacteria could then be addressed either through antibacterial measures or through ordinary nutritive methods. Some later researchers suggested alternative methods of addressing autointoxication: rather than eliminating the harmful ‘organisms’, it might be possible to counteract them with the effects of beneficial bacteria.

What we would now call ‘bacteriotherapy’ is most famously associated with Elie Metchnikoff, a Ukrainian zoologist and microbiologist who was particularly interested in factors that could contribute to human longevity. Influenced by Bouchard’s autointoxication theory, Metchnikoff believed that ailments associated with the aging body, including dementia and neurasthenia, were caused by fermentations and putrefactions produced by colonic microbes, and he saw the colon as a highly problematic, even expendable, part of the human body [54]. He observed, however, that Bulgarian villagers who regularly drank fermented dairy products lived longer than others, and, aware of Pasteur’s work on the effect of lactic acid fermentation in preventing bacterial growth, Metchnikoff theorized that rather than removing the colon or attacking its content, people could instead consume lactic acid as a means of addressing the dangers of putrefactive intestinal bacteria. His views about the role of the intestinal microbiota in longevity and health were published in 1907, and his views on intestinal bacteria in 1910 [55]. His specific comments on ‘fighting microbes with microbes’ were made in 1912 [56]. But he had also made suggestions about ‘introducing useful microbes into the body’ in the form of kefir or soured milk in an earlier book, *The Nature of Man*, in 1903 [57].

Metchnikoff was again working in the context of French medicine: he was positioned at the Pasteur Institute in Paris where he had worked from 1888, and his suggestions were taken up by a broad audience in France. Medical student A. Le Play, for example, in his thesis on ‘Intestinal Poisons’ in 1906, raised the prospect of ‘modifying the intestine’s chemical organisation’ [58]. Journalist Emile Gautier also referred more explicitly in 1907 to the need to combat intestinal poisoning with ‘a police force composed of good microbes’ [59]. He exclaimed that ‘these defensive microbes exist! they are lactic ferments which explain the phenomenal vigour and longevity of Bulgarians: these people, as everyone knows, feed themselves almost exclusively on yoghurt, i.e. soured milk.’

Gautier specifically refers to a French ‘savant’, a Monsieur Chevretin, who isolated the most active of these ferments, the lactic ferment or ‘lactozyme’ B, and dried it before incorporating it into ‘a pastille composed of nutritive substances’. According to Gautier, Chevretin created tablets out of these pastilles ‘which one simply chews with a glass of sugared water to ensure definitive internal health.’ Chevretin’s pastilles are also mentioned in *le Figaro* in 1908 as a means of addressing intestinal intoxication [60] and lactic acid tablets are discussed in the *Gazette médicale de Paris: journal de médecine et Des sciences accessoires* in 1910 [61]. There was therefore a growing sense of excitement in France at this point about the impact of autointoxication on health and the potential means of addressing it.

## Gut microbes reshaping psychiatry

What is most noticeable in the French publications on this area of research in the late nineteenth century is the suggestion that autointoxication is the key concept that will unlock the potential of psychiatry and enable it to move from conjecture and speculation into the realm of facts and science. Chevalier-Lavaure, for example, writing in 1890, acknowledged earlier work in this area but noted that although it would have been possible to group previous findings together, it would not have been possible to form a synthesis, ‘due to the lack of a common basis’. This basis, however, ‘has now been provided by the work of professor Bouchard’, he affirmed [52]. Régis also stated in 1893 that whereas the link between mental disorders and the viscera had long been described by French physicians using the notion of sympathy and other ‘mysterious, scientifically unexplainable causes’, the link could now be explained through autointoxication, that is, ‘the poisoning of the organism and subsequently the brain either by microbes or their secreted outputs, or by toxic substances resulting from the excessive formation of or insufficient elimination of the body’s normal poisons’ [62].

Régis’s first major work on psychiatry, the *Manuel pratique de médecine mentale*, was published in 1884, but his third edition of 1906 was renamed *Précis de Psychiatrie* and was entirely reworked to reflect the changes that had taken place in his discipline. Whereas in 1884, he stated, ‘psychiatry was still an isolated subsection within the medical sciences, existing almost without any change since the clinical discoveries of the first half of the nineteenth century’, after 1892 things had started to change, and since 1893 this medical specialism had been ‘completely transformed’ [31]. Régis was of the opinion that it was ‘the great modern theories of infection and autointoxication’ which had led to the regeneration of psychiatry and brought it into closer contact with medical pathology [31].

**Figure 5.**
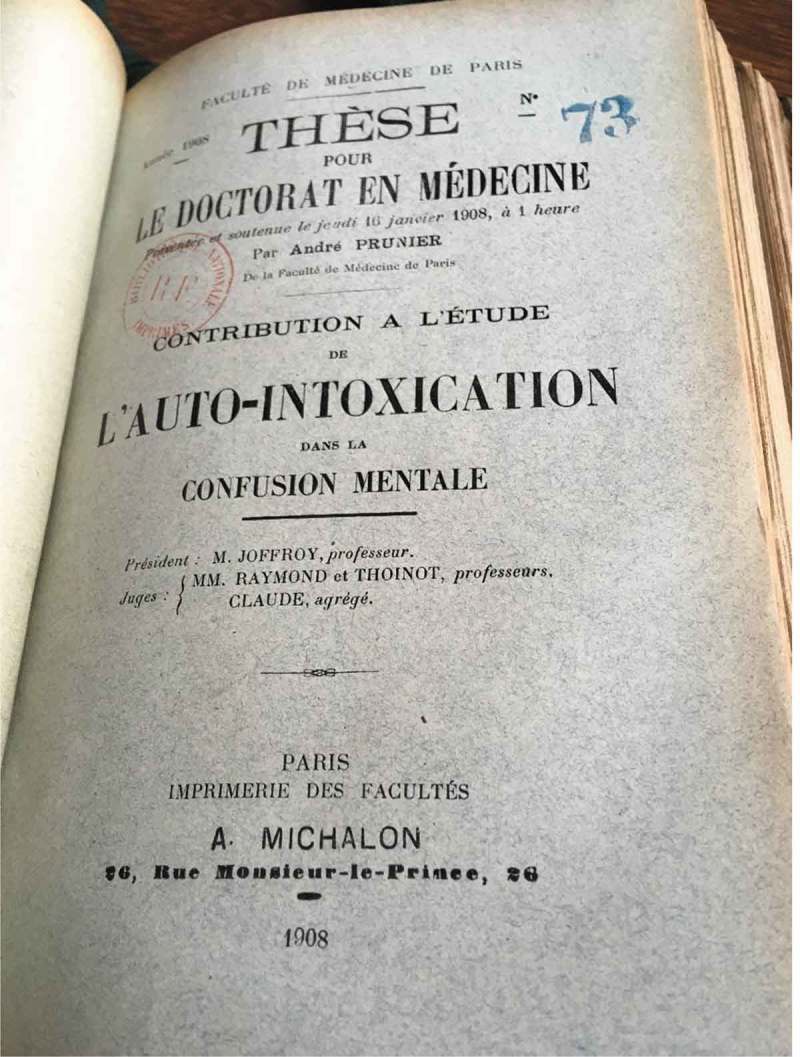
Medical thesis by André Prunier, Bibliothèque interuniversitaire de Santé, Paris, picture taken Manon Mathias, 4 July 2018. Copyright expired.

In a further example, Dr André Prunier, in his 1908 thesis on autointoxication and mental confusion (Figure 5), also described how psychiatry had been ‘transformed’ over the course of the nineteenth century, now becoming a part of medical science: ‘revivified by the application of the fruitful and modern theories of autointoxication and infection’, psychiatry had ceased to exist as ‘a mere chapter within philosophy’ to become, by entering into the fold of general pathology, ‘one of the units within biology’ [51]. He referred to the influence of autointoxication on his field, for instance, by noting the numerous publications which were appearing on the relations between psychosis and nutritional problems. The idea that mental disturbance could be caused by gastro-intestinal problems was, according to Prunier in 1908, ‘no longer in need of demonstration’ [51].

There was therefore a strong sense at the turn of the last century that autointoxication was of serious scientific interest. That psychiatric disturbance that might be caused by intestinal bacteria was considered a crucial factor in bringing psychiatry away from conjecture, hypothesis, and theory into the realm of modern medicine, and concomitantly it was believed that mental conditions could now be understood through biological and physical explanations. Such optimism is palpable today in the proposed applications of microbiome research to the understanding of numerous psychiatric disorders. Bested et al. suggest that one of the reasons behind the discrediting of research into the role of microbes in mental health from the early twentieth century onwards was the influence of Freudian theories about the mind and the growing dominance of psychoanalytical approaches to mental health. Whether one agrees with such a position or not, it is clear that we still have few answers when it comes to successfully treating individuals suffering from conditions such as anxiety, depression, and bipolar disorder, and the situation is becoming increasingly urgent [63]. This is one of the reasons why human gut microbiome studies are stirring such excitement, due to their perceived ability to offer new explanations for the causes of mental health problems and, potentially, new forms of therapy.

As is recognised by many of those now leading the research in this field, caution must be exercised in terms of managing expectations, and few affirm that probiotics are a straightforward method of treatment or that they alone can improve mental health outcomes [64]. Nevertheless, the suggestion made by the major French psychiatrist, Maurice de Fleury, in 1898, that ‘the way forward is now flung open’ and that ‘therapeutic interventions in to ailments of the soul through the action of the physical on the moral is no longer a laughable idea’, might now finally be brought to fruition [65]. By examining the ways in which the theory of autointoxication was beginning to yield fresh understandings of ‘melancholia’ and its treatment at the turn of the last century, we can appreciate the risks, pitfalls, and scepticism involved in undertaking microbial-mental health research, but also the great promise that it holds.
